# RSHash: a fast and space-efficient hash table for *k*-mers

**DOI:** 10.1093/bioinformatics/btag440

**Published:** 2026-08-21

**Authors:** Jonas Schulte-Mattler, Knut Reinert

**Affiliations:** Institutfür Informatik, Freie Universität Berlin, Berlin 14195, Germany; Institutfür Informatik, Freie Universität Berlin, Berlin 14195, Germany; Max-Planck-Institut für Molekulare Genetik, Max-Planck-Gesellschaft, Berlin 14195, Germany

## Abstract

**Summary:**

Large genomic data collections can be viewed as a continuous string of DNA characters. The essential operations for data structures indexing the *k*-mer content of such a string are lookup and locate. Lookup determines whether a query *k*-mer *q* exists in the string and locate returns all locations in the string where *q* is present. High-throughput DNA sequencing generates very many *k*-mer sets of size exceeding billions of characters. In such scenarios, memory consumption and query efficiency pose significant challenges to a data structure supporting the above mentioned queries. To address this problem, we describe a simple, compressed, static data structure for *k*-mers that answers lookup and can be extended for supporting locate. The general scheme follows the use of minimizers like the state-of-the art SSHash. However, instead of using minimum perfect hash functions our solution (RSHash for Rank-Select Hash) relies on bitvectors with rank and select support, a multiple layered minimizer scheme, and a clever buffering strategy. We can show that RSHash is on average 40% and in some cases up to two times faster than SSHash while having the same memory requirements. Indeed we can go as low as 8 bits per canonical 31-mer on a human dataset.

**Availability:**

https://github.com/jonsmcode/rshash.

## 1 Introduction

In recent years many algorithms for analyzing genomic data are based on *k*-mers—short substrings of fixed length *k* over an alphabet of size four. They are used for a wide range of computational methods to solve fundamental tasks in Bioinformatics, including genome and transcriptome assembly, transcript expression quantification, metagenomic classification, structural variation detection, and genotyping (Marchet 2024). These methods build on data structures for representing sets of *k*-mers that support efficient, exact membership lookups. Of particular importance in genomic sequence analysis is the comparison of sequences longer than *k* (e.g. sequencing reads) against large genomic reference datasets via membership queries of all consecutive *k*-mers of a query, which we refer to as streaming lookup. Accordingly, the rapid growth of sequencing data and high-throuput sequencing technologies necessitate compact *k*-mer set data structures that enable fast (streaming) lookups.

The state-of-the-art data structure SSHash (Pibiri 2022) samples *k*-mers of a sequence via its minimizers and stores the minimizers’ positions in a hash table using compact data structures and minimal perfect hash functions (MPHFs). That allows high space efficiency while retaining fast *k*-mer lookups. In this work we follow the minimizer-based approach but replace the use of MPHFs with bitvectors that support rank and select operations and employ a layered multi-minimizer scheme to mitigate the effect of the skewed distribution of minimizers. This results in a *k*-mer hash table that allows streaming lookups 4.5 times faster than the published SSHash and 1.4 times faster than its currently improved version (Pibiri and Patro 2026), while remaining similarly compact, requiring 7 to 10 bits per *k*-mer.

This paper is structured as follows: after we review research on *k*-mer set data structures in Section 2, we introduce our notation and definitions in Section 3. Section 4 presents our data structure. Its performance is evaluated in Section 5. Finally, we propose directions for future work in Section 6.

## 2 Related work

A variety of data structures have been tailored specifically for *k*-mer sets. We refer to the survey of Marchet (2024) and portray only current state-of-the-art static, exact *k*-mer set data structures that align with our approach. They fall into two categories: Burrows-Wheeler Transform (BWT)-based and hashing-based methods.

The Spectral Burrows–Wheeler Transform (SBWT), recently proposed by Alanko *et al.* (2023), represents a *k*-mer set by its de Bruijn graph (dBG) edges, i.e. sequences of character sets succeeding distinct (k−1)-mers. These are succinctly stored using the BWT and data structures that support subset-rank operations, improving on prior work. Sladký *et al.* (2026) broaden dBG-based *k*-mer set representations to *k*-mer superstrings (overlap graphs) which are composed of *k*-mers that may overlap by fewer than k−1 characters. This allows greater data reduction by jointly compressing more overlapping *k*-mers using the BWT. An adapted Masked BWT enables *k*-mer lookups in the compressed superstrings. Currently, it provides the most space-efficient *k*-mer set data structure, called FMSI.

In contrast, hashing-based methods typically offer faster membership queries at the cost of higher space usage. The current line of research builds on MPHFs. The Pufferfish index (Almodaresi *et al.* 2018) employs them to map *k*-mers to positions in a (colored) dBG for answering membership queries. Building on this, Blight (Marchet *et al.* 2021) reduces space consumption by partitioning *k*-mers into buckets via minimizers, using MPHFs within each bucket to locate and verify *k*-mers in a dBG. The aforementioned state-of-the-art SSHash (Pibiri 2022) hashes the positions of minimizers with MPHFs, achieving lower space usage while supporting fast lookups. The data structure is refined further by Pibiri and Patro (2026), making it the fastest compressed *k*-mer set data structure.

In this work we adopt their general method but replace MPHFs with bitvectors and, in doing so, introduce a multiple minimizer scheme to reduce the high frequency tail of the minimizer distributions. We note that a related idea to bound the frequency of minimizers was recently proposed by Alanko *et al.* (2025).

## 3 Preliminaries

We briefly define basic concepts used in this work.

### 3.1 Notation

We denote the natural numbers {n,n+1,…,m} by [*n, m*] and {0,…,n} by *[n]*. Using zero-based indexing, the substring from the character at index *i* up to, but not including, character at j>i is referred to by S[i..j). A *k*-mer is a string of length *k* over alphabet Σ={A,C,G,T}. Let c:Σ→Σ be the complement map A↔T, C↔G. The *reverse complement* $\bar{x}$ of *k*-mer *x* is defined by $\bar{x}[i]=c(x[k-i])$ for i∈[1,k]. A *canonical k*-mer $\hat{x}$ is the minimum of *x* and $\bar{x}$ under an order on Σ.

### 3.2 Minimizers

Let k,m∈N with m≤k and O be a random total order on $\Sigma^{m}$. All canonical *m*-mers of a *k*-mer *x* are $M(x)=\{\hat{x}[i..i+m)|i\in [k-m]\}$. The random *canonical minimizer* of *k*-mer *x* is $m(x)=\min_{O}M(x)$. We define the position of m(x) as $p(x)=\min\{i\in [k-m]|\hat{x}[i..i+m)=m(x)\}$. Note that a random minimizer scheme is usually implemented by choosing the *m*-mery∈M(x) with minimum value h(y) of a uniform hashfunction *h*. In practice, it is sufficient to compute a hash by XOR-ing a *m*-merwith a random value.

Let $T\in \Sigma^{*}$, |T|≥k, n=|T|−k, and K be all *k*-mers in *T*, i.e. K={T[i..i+k)∣,i∈[n]}. We denote by $M=\underset{x\in K}{\cup }m(x)$ the set of all minimizers. The positions of a minimizer *y* in *T* are P(y)={i+p(x)∣y=m(x),x=T[i..i+k),i∈[n]}, the set of all minimizer positions $P=\underset{y\in M}{\cup }P(y)$. We call a minimizer *y frequent* if |P(y)|>t for a threshold t∈N. A *super-k-mer* is a substring *S* of *T* of maximal length s.t. for all *k*-mers *x, y* that are substrings of *S* holds m(x)=m(y). In a random string (k−m+2)/2 consecutive *k*-mers are expected to share the same random minimizer (Zheng *et al.* 2020, Theorem 3). For a set of minimizers M we denote by S(M) all super-*k*-mers s.t. m(x)∈M for any *k*-mer *x* that is a substring of a super-*k*-mer in S(M). For a sequence of strings we denote by M the union of the minimizer sets of all strings for brevity and define P(y) as the positions of minimizer *y* in the concatenation of all strings.

### 3.3 Bitvectors

For bitvector $B\in {\left\{0,1\right\}}^{n}$, $\mathrm{ran}k_{B}(i)=\sum_{j=1}^{i}B[j]$ counts the number of 1 s in *B* up to i∈[n] and $\mathrm{selec}t_{B}(i)=\min\{j|\mathrm{ran}k_{B}(j)=i\}$ gives the position of the *i*-th 1.

### 3.4 Elias-Fano coding

A bitvector of length *U* with *n* 1 s can be encoded with n⌈ log U/n⌉+3n many bits supporting *rank* in O(log(U/n) time (Okanohara and Sadakane 2007).

## 4 RSHash

We now introduce our data structure beginning with the properties of minimizers we exploit, following the approach of Pibiri (2022). Then, we describe the components of our data structure and, finally, propose a layered multi-minimizer scheme to mitigate the penalty of very frequent minimizers.

### 4.1 Sparse hashing

Our development starts with the well-known property of minimizers: consecutive *k*-mers likely have the same minimizer. Since there are (k−m+2)/2 times fewer random minimizers of length *m* than *k*-mers, we index the minimizers to sparsify the dictionary. The smaller *m*, the larger this compression. For example, the human dataset used in the experimental section contains $2.5\cdot 10^{9}$ 31-mers with $3.63\cdot 10^{8}$ random minimizers of length 19, i.e. a compression of 6.9.

### 4.2 Basic data structure layout

Given *p* DNA-strings of total length *N*, the components of our data structure are:

1. *Strings*. The strings are written one after the other in a vector *T* of 2*N* bits, where two bits represent a base in the input. We maintain an Elias-Fano-compressed sorted integer sequence of length *p* to keep track of the endpoints of the strings, which helps avoid detecting sequence-overlapping alien *k*-mers.
2. *Minimizers*. Let M be the set of all canonical minimizers of length *m* encountered in the input. Unlike Pibiri, we mark the occurrence of each encountered minimizer in a bitvector *R* of length $|\Sigma {|}^{m}$. Since *R* is very sparse for sufficiently long *m* it is highly compressible using Elias-Fano coding. The EF-compressed bitvector *R* can also be constructed via a sorted list of minimizers, requiring O(|M|) space during construction, thereby circumventing its initial uncompressed construction that would take space exponential in *m*. The bitvector *R* is preprocessed for rank queries.
3. *Offsets*. We store the positions *P* of the minimizers M in the order induced by *R* in *Offsets* $O\in {\left[N\right]}^{\left|P\right|}$. That means, for the smallest minimizer *r* we first write its offset values P(r) consecutively using $\left\lceil {\;\text{log}\;}_{2}N\right\rceil$ bits for each offset value. Then the offsets for the second smallest minimizer in *R* and so on. We mark the boundaries of the offsets for each minimizer in bitvector $S\in {\left\{0,1\right\}}^{|P|+1}$ with a 1. Bitvector *S* is preprocessed to answer select queries in constant time.

**Figure 1:**
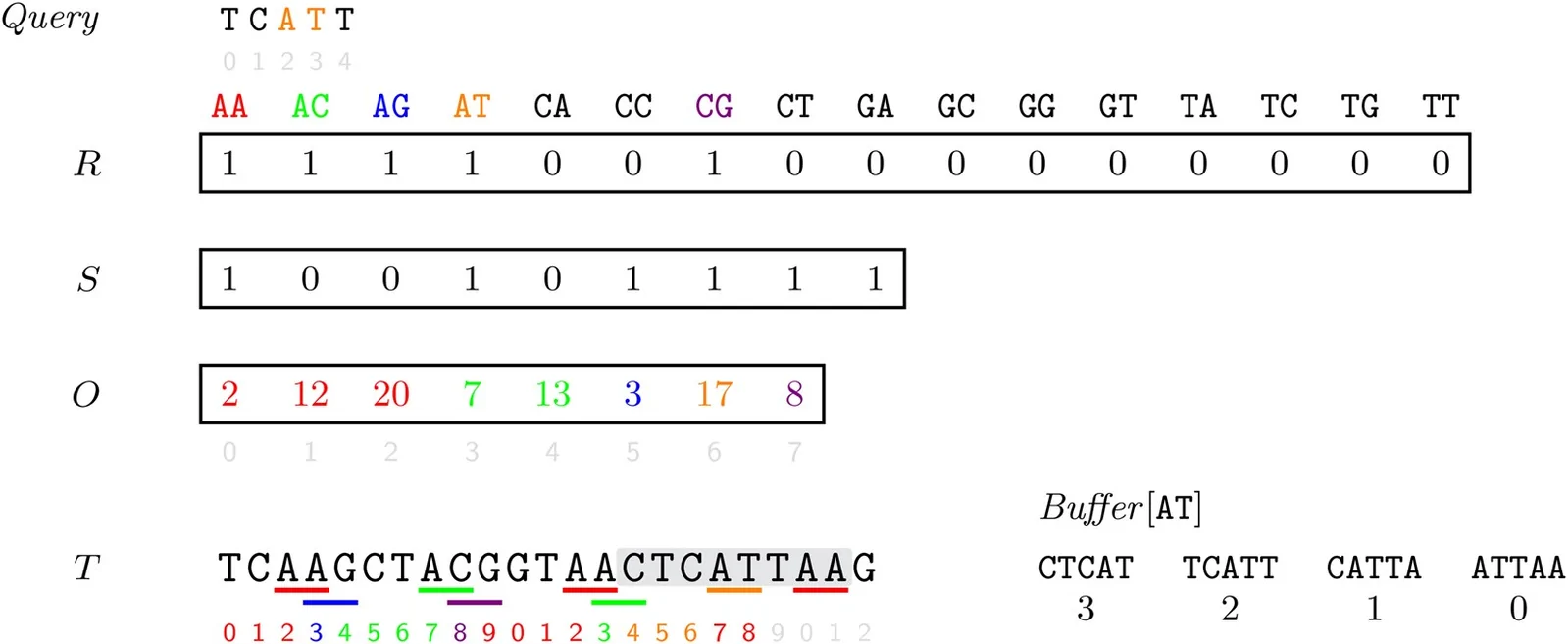
Illustration of the data structure and lookup procedure. We index all 5-mers in *Text T* using lexicographic minimizers of length m=2. The start-position of each 5-mer has the color of its minimizer that is shown with a colored line below the text. The bitvector *R* marks the presence of all minimizers. Bitvector *S* indicates the minimizers’ occurences in the order of *R* and in *Offsets O* all their positions in *T* are saved. To query TCATT we compute its minimizer AT. We find the offsets of AT in *T* (as AT is present in *R*), by first computing its $\mathrm{ran}k_{R}(\mathrm{AT})=4$. Then $\mathrm{selec}t_{S}(4)=6$ is the first position in *Offsets* and $\mathrm{selec}t_{S}(4+1)-1=6$ the last one. For each offset *o* we store all *k*-mers from positions o+m−k to *o* in a *Buffer* from right to left—here all 5-mers from 14 to 17 shaded in gray. Those values are buffered as long as the minimizer does not change. Finally, we can check the presence of TCATT in our buffer. That can be done with one comparison for each occurence of AT: since AT is at position 2 in TCATT, we can only find it at position 2 in the buffer. We ignore the reverse strand in this example for simplicity.

Figure 1 examplifies the data structure’s schema. Let us now see how we can lookup a *k*-mer with bitvectors *R* and *S*, minimizer *Offsets O* and text *T* at hand.

### 4.3 Lookup

We lookup a *k*-mer *x* by first computing its minimizer m(x). If the bit of m(x) is not set in *R*, *x* is not in the data structure (or is a frequent minimizer which will be explained in Section 4). If the bit is set, we compute the minimizer’s rank $r=\mathrm{ran}k_{R}(m(x))$. Then $\mathrm{selec}t_{S}(r)$ tells the index in *Offsets* where the minimizer’s first offset in our text *T* is stored. The last offset is $O[\mathrm{selec}t_{S}(r+1)-1]$ where the next offset belongs to the minimizer with the rank r+1. Now we can check the presence of *x* in *T*. For the moment, let us ignore whether *x* appears in the reverse strand. Let o∈P(m(x)) be an offset of m(x) in *T*. Recall that p(x) is the leftmost position of m(x) in *x*. Then *x* can only appear at position o−p(x) in *T* as m(x) and p(x) are random but fixed. Figure 1 depicts a *k*-mer lookup. Therein, the *k*-mer-buffer serves for streaming lookups as described in the following paragraph.

**Figure 2:**
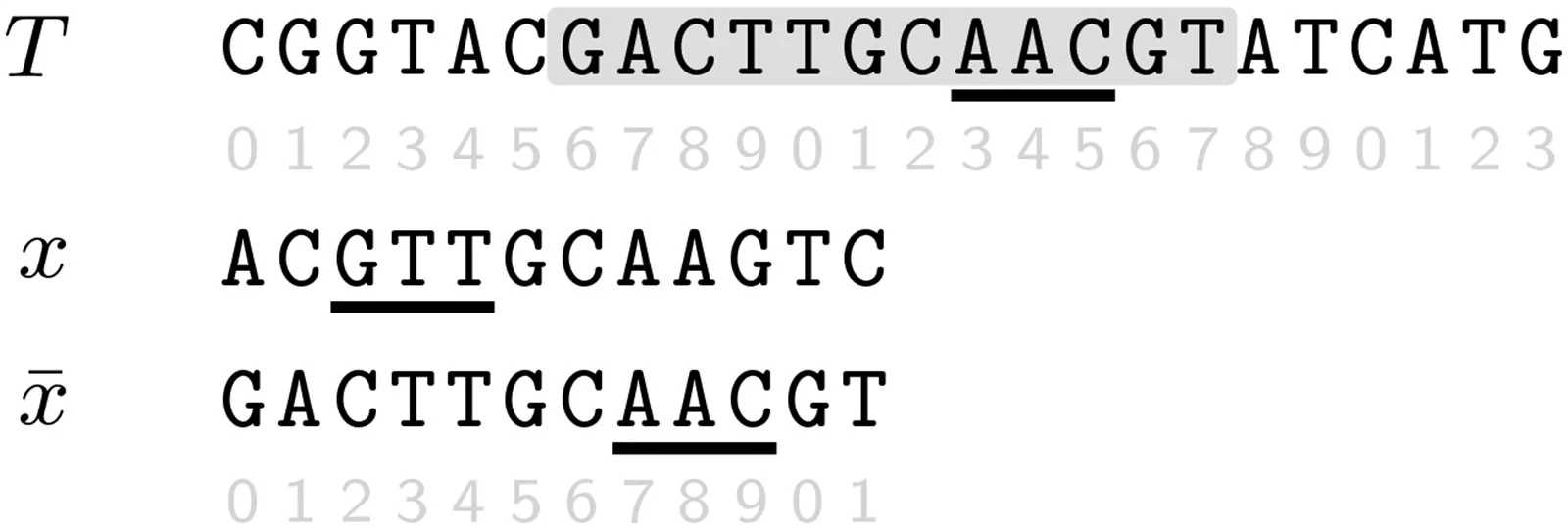
Looking up the reverse complement $\bar{x}$ of 12-mer *x* in text *T*. Its lexicographic canonical minimizer of length 3 is AAC (the reverse complement is GTT). The minimizer’s position in *x* is p(x)=2 and in $\bar{x}$ it is $p(\bar{x})=k-m-p(x)=7$. The *k*-mer $\bar{x}$ is found at position $o-p(\bar{x})=13-7=6$ in *T*.

Considering the presence of the reverse complement $\bar{x}$ in text *T*, we employ canonical minimizers and check for $\bar{x}$ in *T*. Thereby, we have to mind the position where $\bar{x}$ lies relative to *o* in *T*. Observe that the leftmost minimizer at position p(x) in *x* begins at position $p(\bar{x})=k-m-p(x)$ in $\bar{x}$ and vice versa. Hence, *k*-mer $\bar{x}$ can only be present at position $o-p(\bar{x})$ in *T*– see an example in Fig. 2. If the minimizer m(x) appears more than once in a *k*-mer *x*, it is safe to check both cases for the leftmost and rightmost position of m(x) in *x*. Finally, note that we also have to check the sequences’ endpoints not to find alien *k*-mers in the text that overlap two sequences.

### 4.4 Streaming lookup

The lookup procedure can be refined if we wish to lookup all *k*-mers in a string *Q* that is longer than *k* in a streaming fashion. Here, we use the fact that consecutive *k*-mers are likely to share a minimizer and keep all *k*-mers in the text with that minimizer in a buffer located in the thread, i.e. all *k*-mers at positions [o−k+m,o] for each offset *o* of the minimizer (we refer to that procedure with FillBuffer in the following). That allows us to answer lookups for expected (k−m+2)/2 consecutive *k*-mers with the same minimizer efficiently with relevant data in cache (using function LookupBuffer). By keeping track of the minimizer $m^{+}$ whose *k*-mers are currently buffered and of the last encountered minimizer $m^{-}$ that is not in *R*, we can likely answer the positive and negative case for a succeeding *k*-mer fast.

Has a *k*-mer been found at position *p* in our text, we try to elongate the hit in text first, i.e. we check whether the *k*-mer’s following character in the query matches the next character in the text (in the forward or reverse strand respectively—function Extend). The procedure is given in Algorithm 1. We compute the minimizers lazily, i.e. only if a hit is not extended in text.

Algorithm 1 Streaming Lookup

1: hits←0, $m^{+}\leftarrow \infty$, $m^{-}\leftarrow \infty$, found←0, *p*, *strand*

2: **for**  i=0 to |Q|−k  **do** 

3:  x←Q[i..i+k)

4:  **if**  found=1 ∧ EXTENDHIT(p)  **then** 

5:   hits←hits+1, p←p+strand

6:  **else if**  $m(x)=m^{+}$  **then** 

7:   (found,p,strand)←LOOKUPBUFFER(p(x))

8:   hits←hits+found

9:  **else if**  $m(x)\neq m^{-}\wedge \;R[m(x)]$  **then** 

10:   $r\leftarrow \mathrm{ran}k_{R}(m(x))$

11:   $s\leftarrow \mathrm{selec}t_{S}(r)$, $e\leftarrow \mathrm{selec}t_{S}(r+1)$

12:   FillBuffer(*s, e*)

13:   (found,p,strand)←LOOKUPBUFFER(p(x))

14:   $\mathrm{hits}\leftarrow \mathrm{hits}+\mathrm{found},m^{+}\leftarrow m(x)$

15:  **else** 

16:   $m^{-}\leftarrow m(x)$

17:  **end if** 

18: **end for** 

19: **return** *hits*

*Recall: the minimizer of a k-mer x is* m(x)  *and its position in x is* p(x)*. The variable* found∈{0,1}  *indicates if the previous k-mer in query Q is a substring in our text T, p its position, and* strand∈{−1,1}  *its orientation.*

### 4.5 Skewness hashing

The efficiency of the Lookup procedure depends on the number of positions per minimizer, which we refer to as the *frequency* of the minimizer. Since a minimizer can appear multiple times in the input, there is no limit to how large its frequency can grow (other than the trivial upper bound). For instance, as noted by Pibiri, for the human genome, the highest frequency can be as large as $3.6\cdot 10^{4}$ for m=20 (or even larger for smaller values of *m*), which would make a query inspecting all super-*k*-mers extremely slow in practice.

To avoid the burden of these high-frequency minimizers, we exploit another important property of minimizers: the distribution of their frequencies is highly skewed for sufficiently large *m*. This means that most minimizers appear only once, while a relatively small number of them repeat multiple times. For completeness, we report the minimizer frequency distribution on the human dataset used in the experiments in Table 1 along with the percentage of *k*-mers covered by those minimizers.

**Table 1:** Percentage $P_{M}$ of minimizers with frequency at most *f* by minimizer length *m* and percentage $P_{K}$ of 31-mers having those minimizers for the preprocessed human genome without duplicate 31-mers.

f\m	16		17		18		19	
	$P_{M}$	$P_{K}$	$P_{M}$	$P_{K}$	$P_{M}$	$P_{K}$	$P_{M}$	$P_{K}$
1	58.3	20.4	76.1	44.7	87.8	65.8	93.4	78.4
2	77.3	36.1	91.1	64.6	96.3	80.1	98.0	86.7
3	86.5	47.9	95.6	74.0	98.2	84.8	98.9	89.1
64	99.90	92.5	99.95	94.5	99.97	95.7	99.97	96.7

Pibiri (2022) employs an enhanced scheme using MPHFs for frequent minimizers and uses for the human genome a minimizer size of 19 to avoid many frequent minimizers. However, using such a large minimizer size will lead to a relatively large number of super-*k*-mers, since we expect on average a minimizer to be valid for (k−m+2)/2  *k*-mers. Pibiri’s method becomes faster for increasing *m*, but also needs more space. To reduce the size of *Offsets*, we would like to choose *m* relatively small. Hence, we choose to exploit the minimizers’ skewed distribution several times by applying the method outlined in the last section with a second and possibly third hash function. This can easily be done by XOR-ing with random values to compute different hash values.

We construct our index by first computing, as outlined in Section 4, the minimizers using a hash function $h_{1}$ and count how often they occur in hash table $C_{1}$. This count shall not exceed 255 on each level, hence 8 bit are sufficient for the counter. The unfrequent minimizers are sorted to construct the Elias-Fano compressed bitvector $R_{1}$. Before proceeding, we now identify all super-*k*-mers$S(M_{f})$ for minimizers with high frequency (which we call frequent super-*k*-mers), where $M_{f}$ is the set of minimizers with a frequency higher than a specified threshold $t_{1}<256$. Now we simply repeat for all super-*k*-mers in $S(M_{f})$ the first part of construction with a different hash function $h_{2}$. That means we count the occurrences of the minimizers up to a threshold $t_{2}$ and mark the unfrequent ones in a second bitvector $R_{2}$. Now we construct the $S_{2}$ vector and the second offset table. This step can be repeated a third time in RSHash. Of course, this will for the second hash function also result in a skewed distribution with some high-frequency minimizers, but only for a fraction of the text. Finally, for remaining *k*-mers with high frequency minimizers, we employ a fast hash table that stores them directly sacrificing some space in return for a fast lookup which is viable due to our multilevel filtering.

Ultimatly, bounding the number of *k*-mers to verify in text by a constant yields constant lookup time. Note that our Rank-Select scheme allows a quick check for the presence of a minimizer on one of the levels by simply checking a bit which is not easily possible using MPHFs. Our strategy hence allows us to

- Use small minimizers on the first level thereby increasing compression, while
- Reducing frequent minimizers by perturbing the frequent tail of the minimizers’ frequency distributions, and
- Use longer minimizers for *k*-mers with frequent minimizers only.

**Figure 3:**
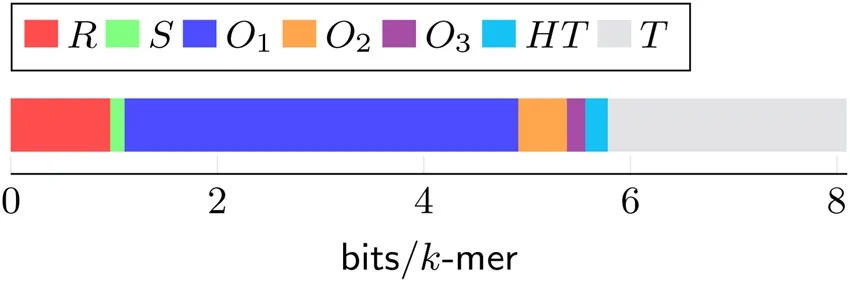
RSHash’s individual components’ space requirement per *k*-mer with multiple levels for the human dataset, i.e. the total space of the bitvectors *R* and *S*, space of Offsets $O_{i}$ for each level *i*, and ultimately the space of hashtable *HT* and text *T*.

To highlight this effect, we analysed the human dataset used in the experimental section. For the thresholds t∈{32,64,128} we store only 31-mers with minimizers whose frequency is less than or equal to *t* in the first level. For all other 31-mers we repeat the construction as described for the second and third level. This has for example for threshold 64 the effect that the *k*-mers that are covered by frequent minimizers are reduced from 5.45% to 2.09% using a second minimizer scheme and ultimately to 0.91% using a third scheme. This saves 5.6 bits per *k*-mer (bpk) of space for expensive *k*-mer hashtable entries (and essentially makes it viable). Table 2 shows this effect for different thresholds and up to three minimizers. Figure 3 depicts the space consumption of RSHash’s compontents for three levels on the human dataset. The Bitvectors $R_{i}$ and $S_{i}$ require approximately 1bpk for all three levels. While minimizer offsets on the first level require 4 bpk, the longer minimizers on levels two and three take only 0.5 bpk such that expensive hash table entries cost only 0.2 bpk. In this experiment adding an additional level slowed negative streaming queries down by 27 to 41 ns per *k*-mer, an average increase of 40%, while positive queries became 13 to 29 ns slower, averaging a 20% increase. The running times are provided in the supplement.

**Table 2:** The percentage $P_{\bar{K}}$ of 31-mers with frequent minimizers and space requirement in bits per *k*-mer (bpk) for the preprocessed human genome using our multiple minimizer scheme with up to three levels and different thresholds *t*.

*t*	128		64		32	
Level	$P_{\bar{K}}$	bpk	$P_{\bar{K}}$	bpk	$P_{\bar{K}}$	bpk
**1**	4.07	10.7	5.45	14.1	7.27	14.0
**2**	1.40	9.21	2.09	9.21	3.03	11.0
**3**	0.53	8.02	0.91	8.50	1.49	9.43

The minimizer lengths $m_{i}$ for levels i∈[1,3] are $m_{1}=17$, $m_{2}=19$, and $m_{3}=21$.

## 5 Experiments

We evaluate the performance of our data structure by comparing it with the closely related state-of-the-art *k*-mer-index SSHash, considering (i) the published version, which we denote SSHash3 (corresponding to GitHub commit 5a13d6a), and (ii) the recently improved release SSHash5 (GitHub commit e65dcb2). That also puts our data structure in perspective to the data structures FMSI and SBWT by their benchmarks against SSHash5 (Pibiri and Patro 2026). There SSHash5 is on average 3x faster for positive streaming queries than SBWT with its plain-matrix variant which constantly requires 10.5bpk and is 12x faster than the very compact FMSI that needs at most 3.5 bpk on our datasets. Both BWT based approaches are more than one order of magnitude slower when accessing a *k*-mer at a given position in the indexed strings than SSHash and RSHash that store the uncompressed input text.

### 5.1 Datasets

We use the same datasets as Pibiri (2022). As noted in their paper, the datasets are: the whole genomes of the atlantic cod (*Gadus morhua*), the common kestrel (*Falco tinnunculus*), the whole GRCh38 human genome (*Homo sapiens*), and a collection of more than 8000 bacterial genomes from Almodaresi *et al.* (2018). Those datasets are preprocessed to filter out duplicate *k*-mers. Namely, a compacted de Bruin Graph is built with BCALM2 (Chikhi *et al.* 2016) whose maximal unitigs (non-branching paths) are extracted using the tool UST (Rahman and Medevedev 2021). This yields the input sequences. Note that this step can be performed more efficiently using GGCAT (Cracco and Tomescu 2023).

### 5.2 Parameter sensitivity

Our multiple minimizer scheme allows two parameters for each level *i*: minimizer-length $m_{i}$ and a threshold for the maximum minimizer frequency. We experimented with up to three levels. Thereby, a genomic dataset’s repetitiveness determines the number of levels necessary to store its *k*-mers efficiently. Minimizer-length $m_{1}$ is chosen within $\left\lceil {\;\text{log}\;}_{4}|T|\right\rceil +c_{1}$ where $c_{1}\in [5]$ and |*T*| is the length of the input text. Futhermore, $m_{2}=m_{1}+c_{2}$ for $c_{2}\in \{1,3,5\}$ and $m_{3}=m_{2}+c_{3}$ for $c_{3}\in \{2,4\}$. Thresholds are selected from {32,64,128}. Both parameters are non-decreasing for increasing levels to have high compression on low levels and a low expected maximum number of super-*k*-mers to verify. For each dataset, we retained the Pareto-optimal configurations, i.e. those not dominated by any other configuration with respect to both memory requirement and query time. From them we choose two configurations per dataset to obtain a compact and a fast version of RSHash. They are depicted in Table 3. Space consumption and running times remain stable across similar parameter settings allowing an easy choice of resonable parameters. Benchmarks for all sets of parameters can be found in the supplementary material.

**Table 3:** Parameter choices for RSHash’s compact and fast version.

		cod	kestrel	human	bacterial
Small	*m*	16,20,24	17,20	17,21,25	18
	*t*	64,64,64	64,128	64,64,64	128
Fast	*m*	16	18	17	19
	*t*	128	128	128	128

Minimizer lengths *m* and thresholds *t* are listed for each level, beginning with the first.

We compare against SSHash, with and without canonical minimizers, to assess a fast and a compact variant. The minimizer-lengths are chosen within $\left\lceil {\;\text{log}\;}_{4}|T|\right\rceil +c$ for c∈[6]. In all experiments *k*-mer length k=31.

### 5.3 Experimental protocol

Our experiments were conducted on an AMD EPYC 9454 server CPU clocked at 2.75 GHz with 1TB of available main memory, using a single core. RSHash is written in C++ and is publicly available, along with the benchmarking procedures (https://github.com/jonsmcode/rshash). It was compiled with GCC 12.2 in release mode under Debian kernel 6.1.0–43, 64 bit. We use implementions of Elias-Fano compressed bitvectors with rank support and bitvectors with select support by Vigna (2008).

### 5.4 Streaming lookup

We use the same sequencing reads as Pibiri (2022) associated with each dataset to perform streaming lookups. These comprise between $2\cdot 10^{6}$ and $3.4\cdot 10^{7}$ reads ranging from 76 to 110 bases. They match 75% to 92% of the *k*-mers in the genomes. Low-hit workloads are generated by permuting the query files which results in less than 1% hits. Both, negative and positive lookups have use cases. Positive lookups are more frequent for example when searching a human probe against a hash table for the the human reference genome. Negative lookups might be expected if *k*-mers are queried against a *k*-mer-table of likely contaminants. Here many queries will return negative results.

**Figure 4:**
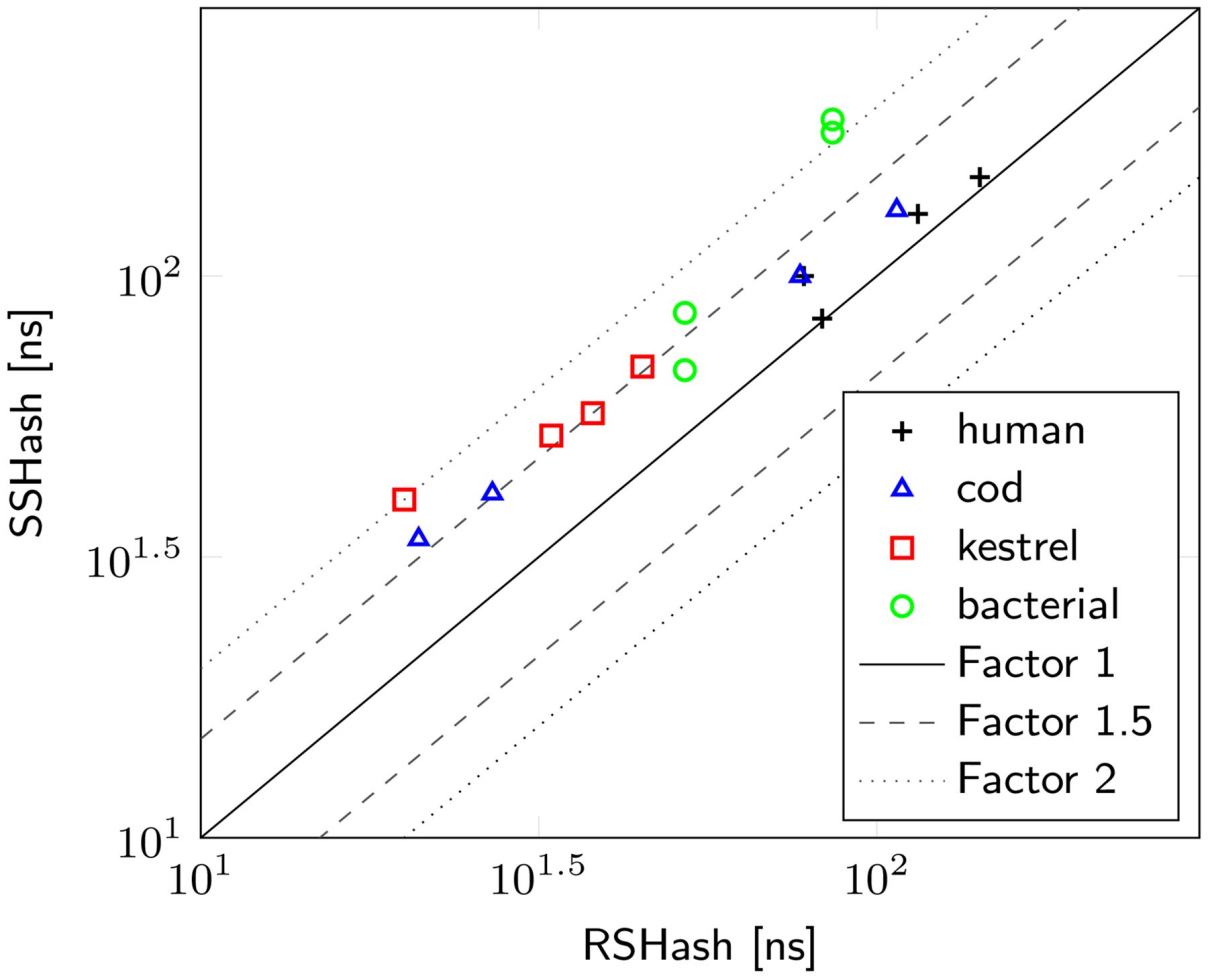
Comparison of SSHash5 and RSHash streaming lookup times given in Table 4. Points above the middle diagonal indicate that RSHash is faster than SSHash. The additional, dotted lines mark a 50% and 100% speedup.

Running times and space requirements are listed in Table 4. RSHash consistently outperforms SSHash5 and is on average 1.4 times and up to two times faster, as shown in Fig. 4, while preserving the same space efficiency. It queries 4.5 times more *k*-mers per second on average and up to 8.5 times more than the published version SSHash3.

**Table 4:** We report the streaming lookup time in ns/*k*-mer and space usage in bits/*k*-mer (bpk) for a compact and fast version of SSHash3, SSHash5, and RSHash.

	**cod**			**kestrel**			**human**			**bacterial**		
	−	+	bpk	−	+	bpk	−	+	bpk	−	+	bpk
SSHash3 fast	200	111	9.2	76	196	8.5	207	296	10.3	242	215	9.2
SSHash5 fast	100	34	9.6	40	57	8.7	100	84	10.1	118	57	9.5
RSHash fast	77	21	9.0	20	38	8.0	78	83	10.7	78	51	9.0
SSHash3 small	478	161	7.5	253	261	7.5	486	344	8.0	733	249	8.2
SSHash5 small	131	41	7.4	52	69	7.2	150	129	7.9	180	68	8.2
RSHash small	107	27	7.4	33	45	7.2	142	115	8.0	86	52	8.0

Low and high hitrates are denoted by − and +.

### 5.5 Lookup

To benchmark negative and positive lookup times of a single *k*-mer, we run at least five rounds of $10^{6}$ lookups of *k*-mers that are either random or randomly sampled from the text (for half of them we compute their reverse complement) until the average lookup time converges. Those are specifically not streaming queries.

The results are reported in Table 5. RSHash’s average lookup time is on par with SSHash5 requiring 0.5μs per *k*-mer in its fast version and 0.6μs in the compact variant across all datasets. Yet, there is a difference between negative and positive lookup times of both data structures. RSHash answers negative lookups 50% faster in its fast version using one level while being 10% slower for positive lookups. In the negative case RSHash mostly has to check a bit in the bitvector *R* and lookup a hash table which is faster than in text verification required using MPHFs. In the positive case, computing a MPHF is slightly cheaper than a rank operation. Note that computing multiple minimizers and checking bitvectors multiple times to tackle repetitive genomic regions with very frequent minimizers comes at the prize of slightly slower lookups. However, the small variant of RSHash is consistently faster, on average 40%, than SSHash that cannot afford to store canonical minimizers.

**Table 5:** Average lookup time in ns/*k*-mer and standard deviation for a compact and fast version of SSHash and RSHash.

	**cod**			**kestrel**			**human**			**bacterial**		
	−	+	bpk	−	+	bpk	−	+	bpk	−	+	bpk
SSHash3 fast	652 ±12	707 ±12	9.2	660 ±3	718 ±0	8.5	816 ±0	892 ±1	10.3	931 ±0	959 ±0	9.2
SSHash5 fast	414 ±1	507 ±2	9.6	435 ±2	500 ±4	8.7	464 ±4	626 ±1	10.1	492 ±1	679 ±1	9.5
RSHash fast	265 ±1	541 ±1	9.0	261 ±1	595 ±1	8.0	360 ±0	701 ±0	10.7	325 ±0	741 ±0	9.0
SSHash3 small	1175 ±1	996 ±0	7.5	1278 ±0	995 ±0	7.5	1538 ±1	1337 ±0	8.0	1727 ±0	1329 ±0	8.2
SSHash5 small	714 ±0	666 ±1	7.4	763 ±1	657 ±1	7.2	875 ±1	936 ±1	7.9	870 ±1	848 ±1	8.2
RSHash small	426 ±3	544 ±0	7.4	362 ±1	609 ±2	7.2	665 ±1	725 ±2	8.0	350 ±1	765 ±1	8.0

Negative and positive lookups are denoted by − and +.

### 5.6 Construction

Table 6 reports time and memory consumption to build RSHash and SSHash on a single core (the fast version). RSHash is built on average 60% faster than SSHash while requiring 40% less memory.

**Table 6:** Time *T* (h: m: s) and memory *M* (GB) required to construct SSHash and RSHash-tables.

	**cod**		**kestrel**		**human**		**bacterial**	
	*T*	*M*	*T*	*M*	*T*	*M*	*T*	*M*
SSHash5	0:58	3.2	2:10	7.5	6:03	14.4	11:31	29.7
RSHash	0:32	2.0	1:24	4.7	3:23	10.4	8:22	26.1
GGCAT	14:06	2.2	26:18	5.0	45:48	11.3	1:40:27	24.0
RSHash gen	0:36	2.6	1:24	4.9	3:55	18.3	28:07	73.3

The tool GGCAT is used to remove duplicate *k*-mers as a necessary preprocessing step for SSHash. RSHash gen constructs the hash table directly given genomes without preprocessing.

Note that Pibiri’s approach relies on preprocessing genomes to remove duplicate *k*-mers. We could not build SSHash with unprocessed, raw genomes as input, which makes the direct use of the offsets in the text cumbersome at best. In contrast, our data structure can be constructed without that preprocessing step. When including the genomes’ preprocessing time for SSHash using the state-of-the-art tool GGCAT in the construction step, RSHash is on average eight times faster to build requiring less memory on all raw genomes except the bacterial dataset. On that dataset RSHash is built almost two times faster but consumes 37% more space. For large collections of similar genomes the construction of a de Bruin graph is clearly reasonable. Using directly the raw genomes has no negative effects on the lookup time for RSHash. They are close to the ones built on the preprocessed datasets. The streaming lookup on the raw genomes is consistently faster than SSHash5 on the compacted de Bruijn graphs across all datasets.

## 6 Future work

Practical improvements include parallelizing the lookup procedure, as well as supporting longer *k*-mers up to length 63 to increase compression. We aim to automate an optimal parameter selection of our multiple minimizer scheme for given input genomes.

Moving on to support locating  *k*-mers, we especially expect our buffering idea in combination with a pipelined search to shine. The construction of our data structure from genomic data without the obstacle of a de Bruijn graph shows promise to efficiently locate  *k*-mers in genomes.

## Supplementary Material

btag440_Supplementary_Data
