# Supplementary material for: RSHash: a fast and space-efficient hash table for k-mers
Source: Bioinformatics. 2026 Aug 21;42(Suppl 2):btag440. doi: 10.1093/bioinformatics/btag440 (PMC13501323; doi:10.1093/bioinformatics/btag440)
Supplement: Supplementary file 1 [file btag440_supplementary_data.pdf]

Bioinformatics, 2026, , 1–1
DOI added during production
Published: Date added during production

RSHash: A fast and space-efficient hash table for k-mers

Jonas Schulte-Mattler[1,*] and Knut Reinert[1,2]

Abstract

This document contains supplementary material.
Contact: j.schulte-mattler@fu-berlin.de

Bitvectors and MPHFs.
To compare Elias-Fano-compressed bitvectors with Minimal Perfect Hash Funktions (MPHFs) which are the key techniques in the respective hashtables RSHash and SSHash (Pibiri, 2022), we report their space usage alongside the running times for the MPHF computation and the respective rank operation for the minimizers in the datasets in Table 1. We benchmarked the EF-compressed bitvectors' rank implementation by Vigna (2008) and the MPHF PTHash (Pibiri and Trani, 2021) used by SSHash. The minimizers have length 17 with a maximum frequency of 64. PTHash is measured for the parameters used in SSHash: partitioned, skewed, D-D encoding, $\alpha = 0.94$, $\lambda = 7$.

Multiple Minimizer scheme.
The effect of our multiple minimizer scheme on space consumption and streaming lookup time for low and high hitrates is reported in Table 2. An additional level slows down negative queries 27 to 41 ns per k-mer, i.e., on average 40%, as negative queries require the computation of l minimizers and l bitvector lookups for l levels while positive queries are 13 to 29 ns slower, averaging a 20% increase.

Parameter sensitivity.
In Figure 1 we show the space consumption and negative streaming query times for all parameter configurations and each dataset. Note that for the datasets KESTREL and BACTERIAL that do not contain many repetitive regions, adding more levels increases running time but does not reduce space consumption – here one level is the best choice. For the datasets HUMAN and COD the use of multiple minimizers reduces the required space notably while the running time increases as expected. Hence, the parameter choice is determined by the input sequences' repetitiveness and the desired time-space-tradeoff that is most sensitive to the number of distinct minimizers and the minimizer length on the first level.

Table 1. Space requirement in bits per minimizer of an Elias-Fano compressed bitvector (EF-BV) and a MPHF together with the running times for a *rank* operation and the computation of the hashfunction in ns.

	Cod		Kestrel		Human		Bacterial	
	bits	ns	bits	ns	bits	ns	bits	ns
EF-BV	11.0	35	9.79	42	9.05	58	8.06	68
MPHF	2.42	14	2.42	29	2.42	35	2.42	45

Table 2. Space requirement in bits per k-mer (bpk) and k-mer streaming lookup times in ns per k-mer for our multiple minimizer scheme with up to three levels and different thresholds t on the preprocessed human genome. Query times for low hitrates are denoted by $-$, for high hitrates by $+$. The minimizer lengths m_i for levels $i \in [1, 3]$ are $m_1 = 17$, $m_2 = 19$, and $m_3 = 21$.

t	128			64			32		
Level	bpk	$+$	$-$	bpk	$+$	$-$	bpk	$+$	$-$
1	10.7	83	78	14.1	71	73	14.0	62	74
2	9.21	112	107	9.21	90	114	11.0	75	111
3	8.02	135	134	8.50	104	145	9.43	88	151

References

G. E. Pibiri. Sparse and skew hashing of k-mers. *Bioinformatics*, 38:I185–I194, 7 2022. ISSN 14602059. doi: 10.1093/bioinformatics/btac245.

G. E. Pibiri and R. Trani. Pthash: Revisiting fch minimal perfect hashing. In *Proceedings of the 44th International ACM SIGIR Conference on Research and Development in Information Retrieval*, SIGIR '21, page 1339–1348. ACM, 2021. doi: 10.1145/3404835.3462849. URL http://dx.doi.org/10.1145/3404835.3462849.

S. Vigna. Broadword implementation of rank/select queries. In *Experimental Algorithms*, volume 5038 of *Lecture Notes in Computer Science*, pages 154–168. Springer, 2008. ISBN 978-3-540-68552-4. doi: 10.1007/978-3-540-68552-4_12.

Received: Date Month Year. **Revised received:** Date Month Year. **Accepted:** Date Month Year
© The Author(s) 2026. Copyright and licence statements to be updated by the publisher during production.

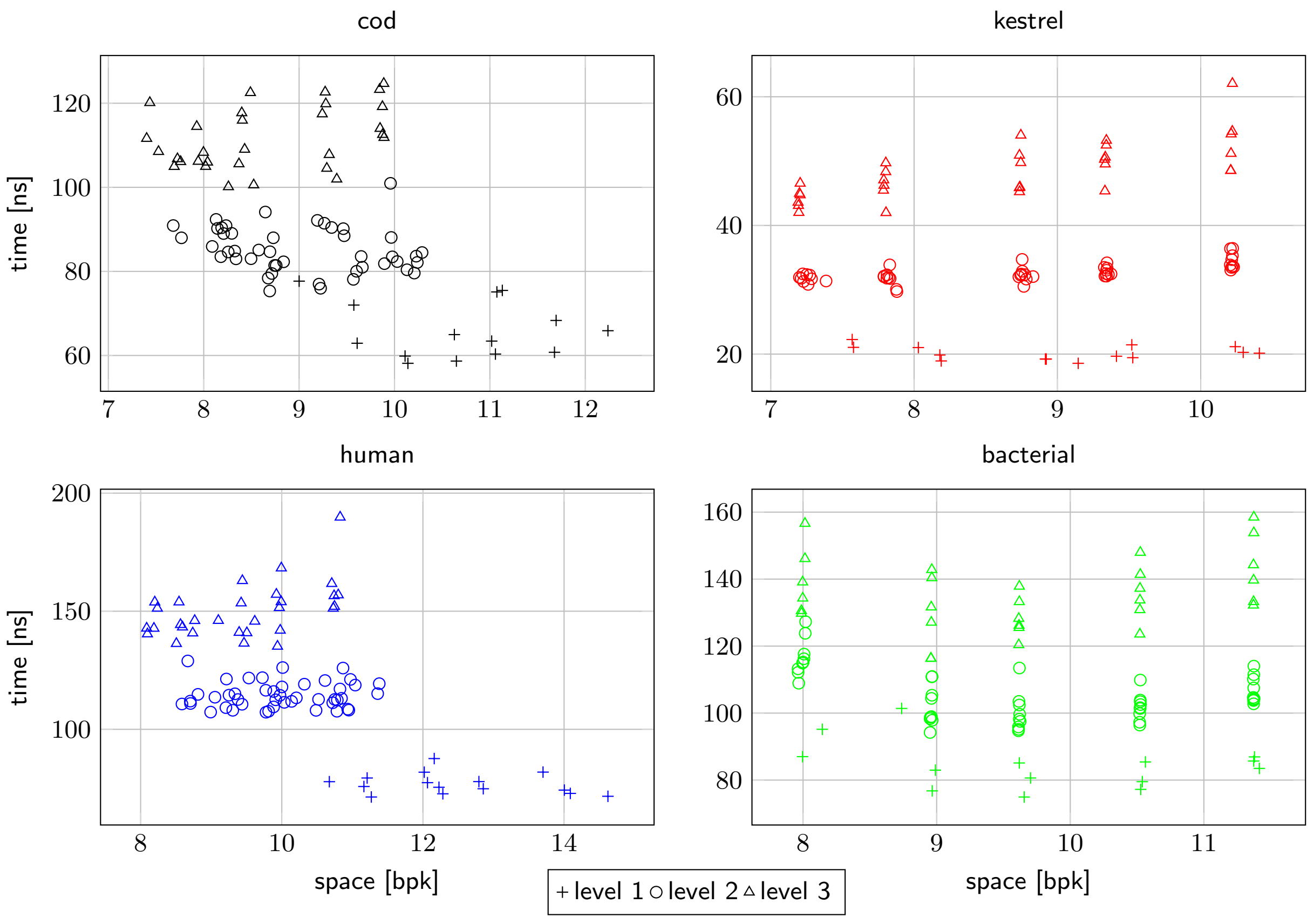

Figure 1 Negative streaming query times and space requirement of RSHash for all parameter choices and for each dataset.
